# Supplementary material for: Is Europe prepared to go digital? making the case for developing digital capacity: An exploratory analysis of Eurostat survey data
Source: PLOS Digit Health. 2022 Feb 17;1(2):e0000013. doi: 10.1371/journal.pdig.0000013 (PMC9931321; doi:10.1371/journal.pdig.0000013)
Supplement: S1 Text — (DOCX) [file pdig.0000013.s001.docx]

**Supplementary Materials**

Van Kessel, R., Wong, BLH., Rubinić, I., O’Nuallain, E., Czabanowska, K. Is Europe prepared to go digital? Making the case for developing digital capacity: an exploratory analysis of Eurostat survey data.

**Text A.** Background information on the analytical frameworks.

**Text B.** Supplementary information on methodology.

**Table A.** Indicators that make up the digital skills measurements of Eurostat.

**Figure A**. Demographic structure of Europe: economically inactive-to-active ratio.

**Text C.** Devices used to access the internet.

**Figure B.** Distribution of devices used to access the internet.

**Text D.** Supplementary reference list.

**Text A.** Background information on the analytical frameworks.

Greenhalgh’s diffusion model

Greenhalgh and colleagues performed an elaborate systematic literature review to identify the factors that influence the spread and sustainenance of innovations in health service delivery and organisations. In doing so, they identified nine relevant dimensions: (1) the innovation itself; (2) the adopter; (3) assimilation; (4) communication and influence; (5) system antecedents; (6) system readiness; (7) outer context; (8) implementation process; and (9) linkage. A visual representation of the model is shown below. Each of these dimensions shows various characteristics that can benefit the uptake of innovation in a new environment. For instance, an innovation is more likely to be taken up in a new system if it has a clear and unquestionable advantage in (cost-)effectiveness, is compatible with the needs and values of the adopter, is simple to use, offers room to experiment, has observable benefits, can be adapted and refined to suit an adopter’s needs, fits in existing infrastructures of the adopter (e.g. is compatible with the hardware that is used by the adopter in case of a software innovation), poses low risks, can make use of existing knowledge to ease the learning curve, and is supported by technical support.

Digital determinants of health

In 1991, Dahlgren and Whitehead conceptualised the now widely used social determinants of health model that divided factors that affected health into five domains (constitutional factors; individual lifestyle factors; social and community networks; living and working conditions; and the general socio-economic, cultural, and environmental conditions) [1]. This model was established at a point where the digital era was still in its infancy. Over time, a need to innovate this model has been expressed and such innovation has been attempted [1]. Rice and Sara innovated the original model by adding a sixth domain on top of the socio-economic, cultural, and environmental conditions: *the virtual world: information and communication technologies* [1]. They briefly explore the impacts of this new domain on physical, mental, and social health. A comprehensive overview of the digital determinants of health was shown later in 2019 by Risling [2], who identified twelve factors that strongly correlate with health in a digital age: (1) income; (2) physical environment; (3) employment; (4) social support and coping skills; (5) gender; (6) health service access; (7) biology/genetics (e.g. age); (8) healthy behaviours; (9) culture; (10) race; (11) childhood; and (12) education/level of literacy.

**Text B.** Supplementary information on methodology.

Demographic Structure in Europe

According to Regulation 1260/2013 on European demographic characteristics, EU Member States send data regarding their population to Eurostat annually. This data includes details on sex, broad age group and NUTS 3, five-year age group and citizenship/country of birth/NUTS 2 & NUTS 3 (under regional data), single age and educational attainment/marital status / broad group of citizenship/broad group of country of birth / NUTS 2 (under regional data), citizenship and broad group of country of birth/country of birth and broad group of citizenship, and population structure statistics: median age of population, proportion of population by various age groups, old age dependency ratio [4].

To further contextualise the data in the main article on economic measures (income, pricing of internet), the demographic structure of economically inactive versus econonomically active people is extracted from this dataset. A full depiction is shown in Figure A below.

**Table A.** Indicators that make up the digital skills measurements of Eurostat.

| **Indicator** | **Competence** | **Sub-categories** |
| --- | --- | --- |
| Information skills | “To identify, locate, retrieve, store, organise and analyse digital information, judging its relevance and purpose” | - Copied or moved files or folders; - Saved files on Internet storage space; - Obtained information from public authorities/services' websites; - Finding information about goods or services; - Seeking health-related information. |
| Communication skills | “To communicate in digital environments, share resources through online tools, link with others and collaborate through digital tools, interact with and participate in communities and networks, cross-cultural awareness” | - Sending/receiving emails; - Participating in social networks; - Telephoning/video calls over the internet; - Uploading self-created content to any website to be shared. |
| Problem solving skills | “To identify digital needs and resources, make informed decisions as to which are the most appropriate digital tools according to the purpose or need, solve conceptual problems through digital means, creatively use technologies, solve technical problems, update one's own and others' competences” | *A – Problem solving*   - Transferring files between computers or other devices; - Installing software and applications (apps); - Changing settings of any software, including operational system or security programs.   *B – Familiarity with online services*   - Online purchases (in the last 12 months); - Selling online; - Used online learning resources; - Internet banking. |
| Software skills | “To create and edit new content (from word processing to images and video); integrate and re-elaborate previous knowledge and content; produce creative expressions, media outputs and programming; deal with and apply intellectual property rights and licences” | *Part A*   - Used word processing software; - Used spreadsheet software; - Used software to edit photos, video or audio files.   *Part B*   - Created presentation or document integrating text, pictures, tables or charts; - Used advanced functions of spreadsheet to organise and analyse data (sorting, filtering, using formulas, creating charts); - Have written a code in a programming language. |

**Figure A.** Demographic structure of Europe: economically inactive-to-active ratio.


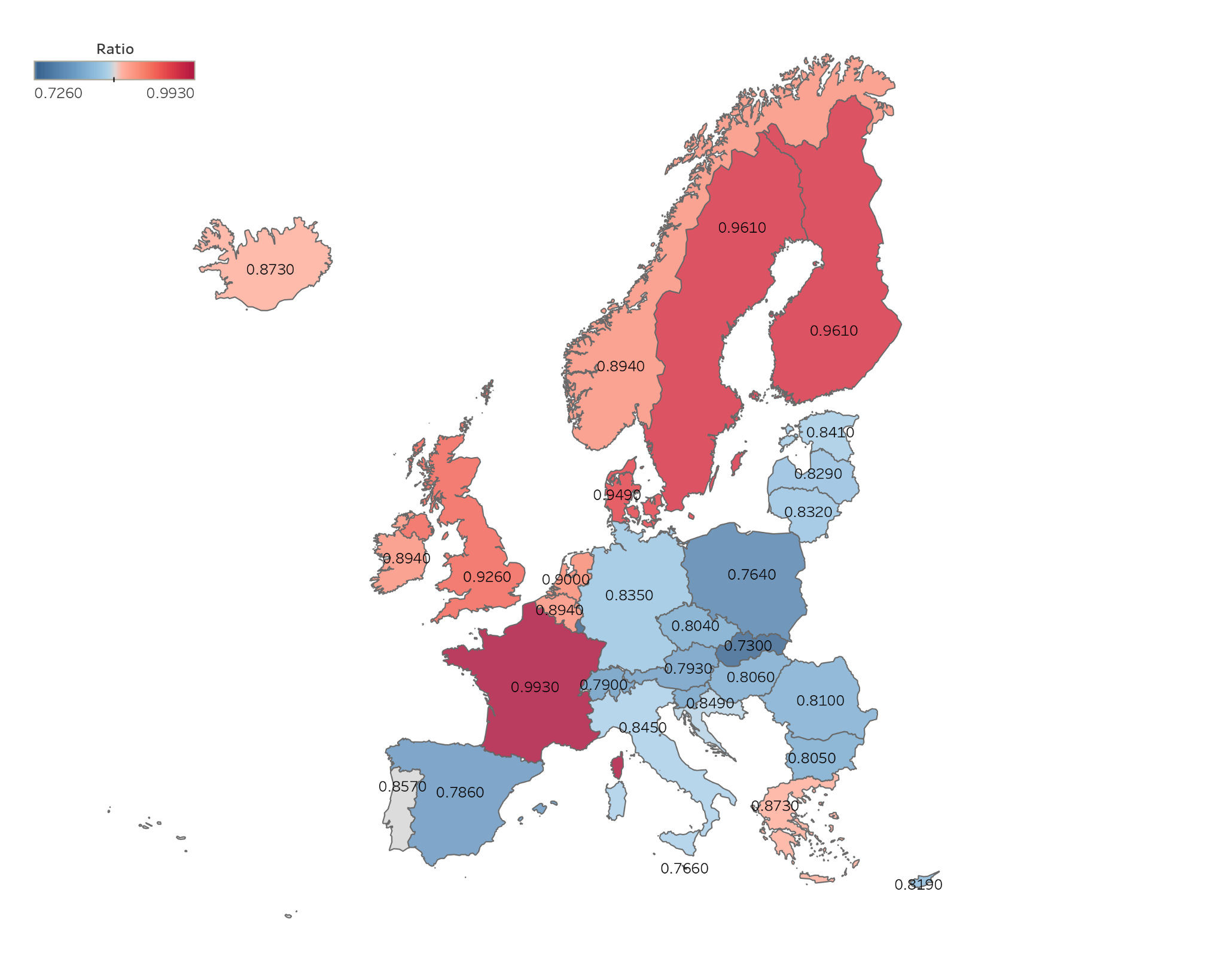


Figure A. A country-level heatmap showing the demographic structure of economically inactive people (ages 0-24 and over 65) versus economically active people (ages 25-65). The maps were generated in Tableau using OpenStreetMap data (© OpenStreetMap Contributors).

**Text C.** Devices used to access the internet.

Descriptive Statistics of Device Usage

Most respondents indicated they used mobile devices to access the internet (80.77%[10.09%]), followed by desktop or laptop (75.53%[12.75%]) and both (61.67%[12.83%]). Similar to the statistics on digital skills, age group 16-24 uses mobile devices more than twice as often to access the internet than 65-74% (97.03%[2.89%] versus 45.93%[22.95%]), desktops or laptops nearly twice as often (87.93%[7.67%] versus 49.47%[23.70%]), and a combination of both nearly three times as often (82.70%[9.44%] versus 28.13%[19.52%]). Mobile device usage to access the internet is more common among females than males in high and medium education (94.03%[3.92%] and 82.80%[11.87%] versus 93.67%[3.92%] and 82.67%[10.10%]), whereas this is reversed in low education level (63.00%[18.83%] for females versus 68.33%[16.66%] for males). Males use desktops or laptops or a combination with mobile devices more often than females. Rural areas score between 8.82 and 12.59 percentage points lower in using devices to access the internet than urban areas. All descriptive statistics are also presented in eTable 2.

Distribution of Devices across Europe

Mobile devices commonly used to access the internet in the age group 16-24 (90-100%), followed by desk- or laptop (66-97%), and a combination of both (53-92%). The age group 25-34 shows similar numbers (86-100%; 60-98%; and 53-92%). Numbers slightly decline in the age group 35-44 (77-99%; 57-96%; and 48-89%), after which they decline more steeply in age 45-54 (63-98%; 51-96%; and 38-86%), age 55-64 (40-97%; 34-97%; and 21-83%), and age 65-74 (13-84%; 16-94%; and 7-66%). Individuals with high education more commonly use mobile devices (85-99%), desk- and laptops (82-99%), and a combination of both (70-93%) to access the internet compared to the medium (61-96%; 52-97%; and 38-85%) and low education strata (33-95%; 24-90%; and 17-79%). Urban regions use mobile devices (71-97%), desk- and laptops (56-96%), and a combination of both (46-89%) to connect to the internet more than rural areas (48-97%; 38-97%; and 28-87%). Students and employed people use mobile devices (92-100% and 68-99%) notably more than unemployed and retired people (42-100% and 23-93%). This trend is also observed in desk- and laptops (74-100% and 61-96% versus 32-94% and 23-93% respectively) and in using a combination of both (67-99% and 50-89% versus 21-86% and 14-66% respectively). eFigure 2 portrays a general and stratified overview of the devices used to access the internet.

**Table B. Devices used to access the internet.**

|  |  |  | **Device Used to Access Internet^1^** | | | | | |
| --- | --- | --- | --- | --- | --- | --- | --- | --- |
|  |  |  | **Mobile** | | **Desktop or Laptop** | | **Desktop or Laptop and Mobile** | |
|  |  |  | **Mean (%)** | **SD (%)** | **Mean (%)** | **SD (%)** | **Mean (%)** | **SD (%)** |
| All Individuals |  |  | 80.77 | 10.09 | 75.53 | 12.75 | 61.67 | 12.83 |
| Age Group | 16-24 |  | 97.03 | 2.89 | 87.93 | 7.67 | 82.70 | 9.44 |
|  | 25-34 |  | 95.57 | 4.03 | 85.30 | 9.67 | 77.07 | 10.56 |
|  | 35-44 |  | 92.20 | 6.09 | 83.20 | 10.06 | 72.70 | 11.03 |
|  | 45-54 |  | 83.50 | 10.08 | 78.10 | 12.82 | 62.37 | 14.11 |
|  | 55-64 |  | 67.13 | 17.42 | 66.77 | 18.59 | 45.13 | 18.18 |
|  | 65-74 |  | 45.93 | 22.95 | 49.47 | 23.70 | 28.13 | 19.52 |
| Level of Education | High |  | 93.83 | 4.08 | 93.10 | 4.69 | 82.53 | 7.00 |
|  |  | Female | 94.03 | 4.31 | 92.30 | 5.04 | 81.13 | 7.50 |
|  |  | Male | 93.67 | 3.92 | 93.97 | 4.32 | 84.43 | 6.66 |
|  |  | 16-24 | 98.87 | 2.42 | 94.22 | 6.47 | 90.57 | 8.10 |
|  |  | 25-54 | 97.57 | 1.70 | 95.17 | 3.47 | 87.97 | 5.60 |
|  |  | 55-74 | 82.17 | 10.88 | 86.60 | 9.32 | 64.43 | 13.56 |
|  | Medium |  | 82.80 | 10.96 | 76.77 | 12.90 | 61.03 | 13.88 |
|  |  | Female | 82.80 | 11.87 | 75.70 | 13.47 | 58.73 | 14.34 |
|  |  | Male | 82.67 | 10.10 | 77.80 | 12.58 | 63.07 | 13.53 |
|  |  | 16-24 | 97.90 | 2.16 | 89.43 | 7.00 | 84.47 | 8.83 |
|  |  | 25-54 | 90.53 | 6.76 | 80.90 | 11.94 | 67.17 | 13.26 |
|  |  | 55-74 | 62.70 | 20.28 | 64.60 | 20.31 | 39.87 | 19.38 |
|  | Low |  | 65.70 | 17.49 | 56.80 | 19.90 | 43.63 | 18.37 |
|  |  | Female | 63.00 | 18.83 | 53.27 | 21.00 | 39.87 | 19.01 |
|  |  | Male | 68.33 | 16.66 | 60.70 | 19.34 | 47.93 | 18.41 |
|  |  | 16-24 | 95.79 | 4.64 | 84.69 | 10.14 | 78.72 | 12.51 |
|  |  | 25-54 | 73.87 | 18.17 | 58.20 | 19.57 | 43.37 | 18.46 |
|  |  | 55-74 | 37.83 | 24.55 | 36.50 | 25.49 | 19.43 | 17.63 |
| Urban Status | Urban |  | 84.34 | 7.59 | 79.93 | 10.01 | 68.00 | 11.07 |
|  | Suburban or Town | | 80.72 | 10.15 | 74.76 | 13.46 | 60.38 | 12.98 |
|  | Rural |  | 75.52 | 13.28 | 70.45 | 16.92 | 55.41 | 15.86 |
| Employment Status | Employed |  | 89.83 | 6.20 | 83.63 | 9.93 | 71.50 | 10.89 |
|  | Unemployed |  | 77.29 | 14.56 | 67.04 | 15.45 | 51.43 | 14.83 |
|  | Retired |  | 56.63 | 19.78 | 55.03 | 20.40 | 33.97 | 16.72 |
|  | Student |  | 97.90 | 2.25 | 92.07 | 5.90 | 87.43 | 7.32 |
| Income Level | First Quartile |  | 62.65 | 19.34 | 55.38 | 19.87 | 41.12 | 16.91 |
|  | Second Quartile |  | 73.77 | 13.89 | 68.19 | 16.19 | 50.67 | 15.13 |
|  | Third Quartile |  | 84.58 | 10.10 | 80.42 | 12.36 | 65.03 | 13.44 |
|  | Fourth Quartile |  | 92.12 | 5.95 | 89.69 | 7.58 | 79.19 | 8.76 |
| ^1^ Data from the 2018 survey | |  |  |  |  |  |  |  |

**Figure B.** Distribution of devices used to access the internet.


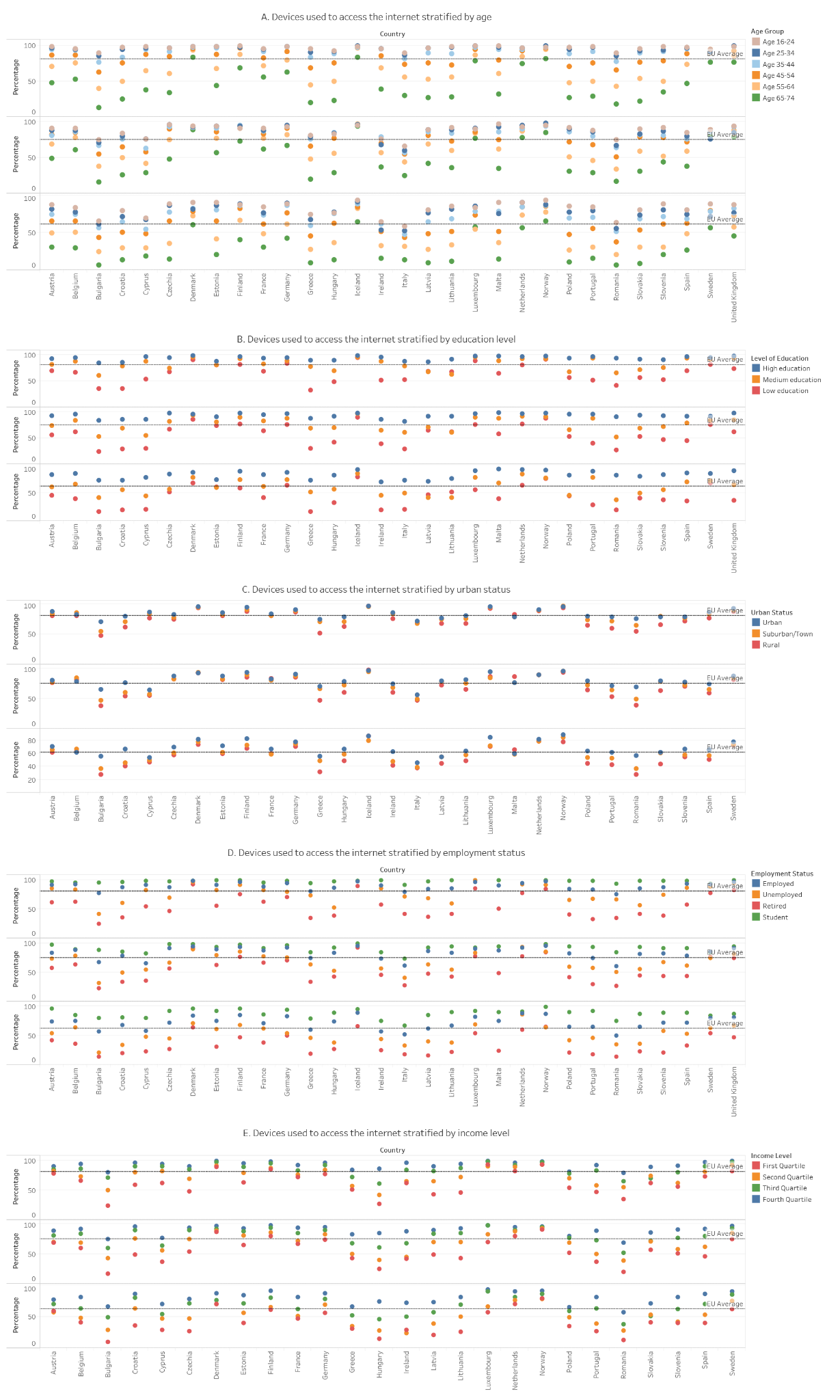


Figure B. Scatterplots to indicate what devices are used to access the internet, divided by age category (Figure B-A), education level (Figure B-B), urban status (Figure B-C), employment status (Figure B-D), and income level (Figure B-E). The upper plot depicts the use of mobile devices only, the middle plot desk- or laptops, and the lower a combination of mobile and desk- or laptop.

**Text D**

1. Rice L, Sara R. Updating the determinants of health model in the Information Age. Health Promot Int. 2019 [cited 2021 Feb 26];34:1241–9. Available from: https://academic.oup.com/heapro/article/34/6/1241/5095999

2. Risling T. Beyond the Divide: Exploring the Digital Determinants of Health. In: Canada Health Infoway: Infoway Partnership Conference. 2019. Available from: https://www.infoway-inforoute.ca/en/component/edocman/supporting-documents/partnership/3770-ipc2019-day02-1040-1110-tracie-risling-en-pdf?Itemid=101

3. European Commission. Communication from the Commission to the European Parliament, the Council, the European Economic and Social Committee and the Committee of the Regions: A New Skills Agenda for Europe - Working together to strengthen human capital, employability, and compe. COM/2016/0381 final 2016. Available from: https://eur-lex.europa.eu/legal-content/EN/TXT/?uri=CELEX:52016DC0381

4. Eurostat. Metadata Population Structure. 2019 [cited 2021 May 27]. Available from: https://ec.europa.eu/eurostat/cache/metadata/en/demo_pop_esms.htm
