# Supplementary material for: Is Europe prepared to go digital? making the case for developing digital capacity: An exploratory analysis of Eurostat survey data
Source: PLOS Digit Health. 2022 Feb 17;1(2):e0000013. doi: 10.1371/journal.pdig.0000013 (PMC9931321; doi:10.1371/journal.pdig.0000013)
Supplement: Attachment — Submitted filename: Response to Reviewers.docx [file pdig.0000013.s002.docx]

Journal Requirements:

**1. Please update the completed 'Competing Interests' statement, including any COIs declared by your co-authors. If you have no competing interests to declare, please state "The authors have declared that no competing interests exist". Otherwise please declare all competing interests beginning with the statement "I have read the journal's policy and the authors of this manuscript have the following competing interests:"**

Thank you for this comment. We have adjusted the Competing Interests statement to adhere to the journal standards.

**2. If you did not receive any funding for this study, please simply state: “The authors received no specific funding for this work.”**

Thank you for this comment. We have adjusted the funding statement accordingly.

**3. Please note that your Data Availability Statement is currently missing a direct link to access each database. If your manuscript is accepted for publication, you will be asked to provide these details on a very short timeline. We therefore suggest that you provide this information now, though we will not hold up the peer review process if you are unable.**

Thank you for this comment. We have added a Data Availability Statement and added the URL to the database website.

**4. Please provide separate figure files in .tif or .eps format only and remove any figures embedded in your manuscript file. Please ensure that all files are under our size limit of 20MB.**

**Once you've converted your files to .tif or .eps, please also make sure that your figures meet our format requirements**

**For more information about how to convert your figure files please see our guidelines:** [**https://journals.plos.org/digitalhealth/s/figures**](https://journals.plos.org/digitalhealth/s/figures)

**5. We have noticed that you have uploaded supporting information but you have not included a list of legends. Please add a full list of legends for all supporting information files (including figures, table and data files) after the references list.**

Thank you for this comment. We have added a list of legends for supporting information files.

**6. Please provide us with a direct link to the base layer of the map used in Figures 2, 3, and supplementary eFigure 1, and ensure this location is also included in the figure legend.**

**Please note that, because all PLOS articles are published under a CC BY license (**[**creativecommons.org/licenses/by/4.0/**](http://creativecommons.org/licenses/by/4.0/)**), we cannot publish proprietary maps such as Google Maps, Mapquest or other copyrighted maps. If your map was obtained from a copyrighted source please amend the figure so that the base map used is from an openly available source.**

**Please note that only the following CC BY licences are compatible with PLOS licence: CC BY 4.0, CC BY 2.0 and CC BY 3.0, meanwhile such licences as CC BY-ND 3.0 and others are not compatible due to additional restrictions. If you are unsure whether you can use a map or not, please do reach out and we will be able to help you.**

**The following websites are good examples of where you can source open access or public domain maps:**

*** U.S. Geological Survey (USGS) - All maps are in the public domain. (**[**http://www.usgs.gov**](http://www.usgs.gov/)**)**

*** PlaniGlobe - All maps are published under a Creative Commons license so please cite “PlaniGlobe,** [**http://www.planiglobe.com**](http://www.planiglobe.com/)**, CC BY 2.0” in the image credit after the caption. (**[**http://www.planiglobe.com/?lang=enl**](http://www.planiglobe.com/?lang=enl)**)**

*** Natural Earth - All maps are public domain. (**[**http://www.naturalearthdata.com/about/terms-of-use/**](http://www.naturalearthdata.com/about/terms-of-use/)**)**

Thank you for this comment. The maps were drawn using licensed Tableau software, which makes use of OpenStreetMap data. We have added the details to the data availability heading.

**Reviewer #1: I have reviewed this informative study. It is a matter of great pleasure to assess this informative study. I believe the authors have examined a good research topic entitled, “Is Europe prepared to go digital? Making the case for developing digital capacity: an exploratory analysis of Eurostat survey data.”**

**In my assessment, the writing of this article looks sounds good with a creative research topic. I have some suggestions to the authors to improve the quality of this article. Overall, it a good article that offers useful insight for the scholars.**

**I want to accept your study for publication after minor changes as suggested. Before endorsing your study for publication, you need to work on my suggestions. The suggested articles are published in leading SSCI journals. By following these studies, your article will be improved.**

**Abstract of the study**

**The abstract is proper and shows good connection of the study. I advise the authors to recheck the abstract and fix minor grammar errors. The abstract must reflect high quality, as it the "FACE" of the study.**

Thank you for this comment. We have reviewed and clarified the abstract.

In-text edits:

Page 2: "...is a sample of 147,531 households and 197,631 individuals..."

Page 2: "...to ensure they can benefit optimally, equitably, and sustainably..."

**English level**

**I suggest the authors to focusing on checking some typo errors to make it easy to understand for the readership of the journal.**

**Introduction section**

**I strongly advise the authors improve introduction according to suggested articles in the introduction section. These research articles have identified health-related topics. I believe it will improve the quality of your work. I strongly suggested them to improve this section a bit more. I advise authors to revisit their literature section of the recommended studies and cite these studies to enhance your research study's quality to reach scientific merit for publication.**

**Azizi, M. R., Atlasi, R., Ziapour, A., & Naemi, R. (2021). Innovative human resource management strategies during the COVID-19 pandemic: A systematic narrative review approach. Heliyon, 6(12).**

**Abbas, J., Aqeel, M., Jaffar, A., Nurunnabi, M., and Bano, S. (2019). "Tinnitus perception mediates the relationship between physiological and psychological problems among patients." Journal of Experimental Psychopathology, 10(3), 2043808719858559.**

**Aqeel, M., et al., The Influence of Illness Perception, Anxiety and Depression Disorders on Students Mental Health during COVID-19 Outbreak in Pakistan: A Web-Based Cross-Sectional Survey. International Journal of Human Rights in Healthcare, 2020. 14.**

**Abbas, J., Wang, D., Su, Z., & Ziapour, A. (2021). The Role of Social Media in the Advent of COVID-19 Pandemic: Crisis Management, Mental Health Challenges and Implications. Risk Manag Healthc Policy, Volume 14, 1917-1932. doi:10.2147/rmhp.S284313**

**Su, Z., McDonnell, D., Wen, J., Kozak, M., Šegalo, S., . . . Xiang, Y.-T. (2021). Mental health consequences of COVID-19 media coverage: the need for effective crisis communication practices. Globalization and Health, 17(1), 4. doi:10.1186/s12992-020-00654-4**

Thank you for your comments. We included some of the proposed literature to strengthen the introduction of this manuscript.

In-text edits:

Page 3: Inserted "during the pandemic [16-18]."

**Literature sections**

**I recommend the authors add suggested articles in the literature section. These research articles have identified health-related topics. I believe it will improve the quality of your work. I advise authors to revisit their literature section of the recommended studies to enhance your research study's quality to reach scientific merit for publication.**

**I want to see publish this creative work after some corrections. I have endorsed this study as; it deserves the merit for publication. However, I suggest the authors make minor corrections according to my advice. The authors add the latest citations about infectious disease. Please read the suggested studies and execute them in the introduction, literature, and method sections. How social media and internet use among students is helpful. Add few lines in the introduction and literature sections.**

**NeJhaddadgar, N., Ziapour, A., Zakkipour, G., Abolfathi, M., & Shabani, M. (2020, 2020/11/13). Effectiveness of telephone-based screening and triage during COVID-19 outbreak in the promoted primary healthcare system: a case study in Ardabil province, Iran. Journal of Public Health.** [**https://doi.org/10.1007/s10389-020-01407-8**](https://doi.org/10.1007/s10389-020-01407-8)

**Abbas, J. (2021, 2021/02/23/). Crisis management, transnational healthcare challenges and opportunities: The intersection of COVID-19 pandemic and global mental health. Research in Globalization, 100037.** [**https://doi.org/10.1016/j.resglo.2021.100037**](https://doi.org/10.1016/j.resglo.2021.100037)

**Maqsood, A., Abbas, J., Rehman, G., & Mubeen, R. (2021, 2021/11/01/). The paradigm shift for educational system continuance in the advent of COVID-19 pandemic: Mental health challenges and reflections. Current Research in Behavioral Sciences, 2, 100011.** [**https://doi.org/10.1016/j.crbeha.2020.100011**](https://doi.org/10.1016/j.crbeha.2020.100011)

**Shuja, K. H., Aqeel, M., Jaffar, A., & Ahmed, A. (2020, Spring). COVID-19 Pandemic and Impending Global Mental Health Implications. Psychiatr Danub, 32(1), 32-35.** [**https://doi.org/10.24869/psyd.2020.32**](https://doi.org/10.24869/psyd.2020.32)

**Su, Z., Wen, J., Abbas, J., McDonnell, D., Cheshmehzangi, A., Li, X., Ahmad, J., Segalo, S., Maestro, D., & Cai, Y. (2020, Dec). A race for a better understanding of COVID-19 vaccine non-adopters. Brain Behav Immun Health, 9, 100159.** [**https://doi.org/10.1016/j.bbih.2020.100159**](https://doi.org/10.1016/j.bbih.2020.100159)

Thank you for these comments. However, this manuscript discusses digital skills and literacy. The proposed sources are from different disciplines, namely mental health or infectious diseases. While they are an interesting read, we do not see a valid reason to include these sources in the current manuscript.

**Materials and Methods**

**This section indicates how you arranged your article. You can see the suggested study and improve your method section.**

**Abbas, J., Aqeel, M., Abbas, J., Shaher, B., A, J., Sundas, J., and Zhang, W. (2019). "The moderating role of social support for marital adjustment, depression, anxiety, and stress: Evidence from Pakistani working and nonworking women." J Affect Disord, 244, 231-238.**

**Local Burden of Disease, H. I. V. C. (2021). Mapping subnational HIV mortality in six Latin American countries with incomplete vital registration systems. BMC Medicine, 19(1), 4. doi:10.1186/s12916-020-01876-4**

**Abbas, J., Aman, J., Nurunnabi, M., & Bano, S. (2019). The Impact of Social Media Learning Behavior for Sustainable Education: Evidence of Students from Selected Universities in Pakistan. Sustainability, 11(6), 1683.**

**Abbasi, K. R., Abbas, J., and Tufail, M. (2021). "Revisiting electricity consumption, price, and real GDP: A modified sectoral level analysis from Pakistan." Energy Policy, 149, 112087.**

**Yoosefi Lebni, J., Abbas, J., Khorami, F., Khosravi, B., Jalali, A., and Ziapour, A. (2020). "Challenges Facing Women Survivors of Self-Immolation in the Kurdish Regions of Iran: A Qualitative Study." Frontiers in psychiatry, 11, 778.**

Thank you for this comment. We believe the shortcomings you are hinting at are already addressed by the comments of reviewer 3. We would like to invite you to read our responses to their comments as a response to this comment.

**Results**

**The results section looks good. The authors can refine it by removing some typo errors.**

Thank you for this comment. We have reviewed the results and removed some grammer and spelling mistakes.

**Discussion section**

**Briefly discuss the contribution to the scientific literature. Add few lines on contribution of this study how results are insightful for academic purpose. Improve this section. Please see suggested studies in this section.**

**Su, Z., McDonnell, D., Cheshmehzangi, A., Abbas, J., Li, X., & Cai, Y. (2021). The promise and perils of Unit 731 data to advance COVID-19 research. BMJ Global Health, 6(5), e004772.** [**https://doi.org/10.1136/bmjgh-2020-004772**](https://doi.org/10.1136/bmjgh-2020-004772)

**Abbas, J. (2020, Autumn - Winter). The Impact of Coronavirus (SARS-CoV2) Epidemic on Individuals Mental Health: The Protective Measures of Pakistan in Managing and Sustaining Transmissible Disease. Psychiatr Danub, 32(3-4), 472-477.** [**https://doi.org/10.24869/psyd.2020.472**](https://doi.org/10.24869/psyd.2020.472)

**Yoosefi Lebni, J., Abbas, J., Moradi, F., Salahshoor, M. R., Chaboksavar, F., Irandoost, S. F., Nezhaddadgar, N., & Ziapour, A. (2020, Jul 2). How the COVID-19 pandemic effected economic, social, political, and cultural factors: A lesson from Iran. International Journal of Social Psychiatry, 20764020939984.** [**https://doi.org/10.1177/0020764020939984**](https://doi.org/10.1177/0020764020939984)

**Abbas, J., Mubeen, R., Iorember, P. T., Raza, S., & Mamirkulova, G. (2021). Exploring the impact of COVID-19 on tourism: transformational potential and implications for a sustainable recovery of the travel and leisure industry. Current Research in Behavioral Sciences, 2, 100033.**

Thank you for this comment. We have added a few lines at the start of the discussion that highlight the key takeaways from this article. That said, the discussion has also been adjusted based on the comments of other reviewers. We believe these comments also address your concerns.

In-text edits:

Page 11: Inserted "The findings of this article highlight that Europe cannot be considered a monolith when it comes to internet access or digital skills. In other words – while Europe may outperform other world regions on average in internet access and digital skills – substantial in- and cross-country inequalities still persist."

**Implications**

**Add this separate heading and discuss implications**

Thank you for this comment. The implications of this research are discussed under the heading 'Recommendations for Policy and Governance'. To further clarify this, we have amended the heading. We also added a separate heading for the implications for future research.

In-text edits:

Page 13: Inserted "Implications for Future Research"

Page 13: Inserted "This study first and foremost highlighted that Europe cannot be considered a monolith when it comes to internet access and digital literacy. While we advocate for country-level comparisons, we believe splitting Europe into five regions may be the most valuable for accurate comparisons in case a study design requires countries to be pooled into clusters (North-West, North-East, Center, South-West, and South East). This ensures countries with similar internet access and digital skill levels are clustered, thus removing the bias they would introduce if these regions were lumped together. Further research into the cultural and motivational factors is also warranted to further the understanding of why certain areas in Europe experience difficulties in connecting to the Internet and developing digital skills. Finally, this article is only a single time point in determining the digital capacities of Europe. Therefore, we recommend that this article be replicated using newer data once available in order to establish longitudinal trends."

Page 14: Inserted "Implications and Recommendations for Policy and Governance"

**Conclusion**

**Make a separate heading for this section. It should present a good picture of the study. I want to see this manuscript published as it has presented a good research topic, although it needs minor corrections, which can be fixed in the revised version. Pay attention of English quality to reach scientific merit. I accept and endorse this manuscript for publication after the suggested minor corrections.**

Thank you for this comment. We have amended the conclusion to better finish this manuscript.

In-text edits:

Page 15: Inserted "Based on the current findings, we conclude that..."

Page 15: Inserted "..., showcasing large in- and cross-country discrepancies in internet access and digital skills."

**Figures & Tables**

**I suggest to add some figures and more tables in this study**

Thank you for this comment. After careful consideration and the knowledge that this study already includes five figures and one table, we did not deem it necessary to add more figures without a compelling reason. The Supplementary Materials include more figures, specifically on the devices that are used to access the internet. However, this data is not central to the discussion, hence our decision to keep it in the supplementary material.

**Reviewer #2: The data are well presented and support the discussions/conclusions as well as recommendations for policy and governance. The tables as well as figures included in the paper are clear and easy to understand. The analysis clearly shows that there are demographic and other factors affecting access to and capacities in use of digital tools and applications.**

Thank you for this kind review.

**Reviewer #3: p.4 The Community Survey and Data Access**

**This paragraph is too short and lack info on important characteristics of the survey:**

**- How were households selected?**

**- The sentence "the survey data [...] represents approximately 75% of the population between the ages of 16 and 74" is ambiguous. A sample size of about 200000 individuals is by all means a large sample size.**

Thank you for this comment. We have expanded the background information on the community survey and included what data sampling methods were used per country. It was impossible for us to elaborate how households and individuals were precisely selected per country, as each country had its own mechanisms on they recruited their participants. This information is all present in the reference however.

In-text edits:

Pages 4-5: inserted "The community survey on ICT usage in households and by individuals provides a set of variables that, when combined, measure an individual’s level of digital skills. They are based on the 2016 Digital Competence Framework of the European Commission in which key skills relating to digital technologies are set out [[8]](https://www.zotero.org/google-docs/?0wFFcz). Proficiency in digital skills is divided into four levels: above basic digital skills, basic digital skills, low digital skills, and no digital skills. The level of digital skills is determined by the reported outcomes in four domains: information skills, communication skills, problem-solving skills, and software skills (for content manipulation). Further breakdown of the domains into their respective subcategories is provided in eTable 1 in the Supplementary Material.

Individuals with above basic digital skills score “above basic” in all four domains; individuals with basic digital skills score at least one “basic” but no “no skills” in all four domains; individuals with “low” digital skills (i.e. missing some type of basic skills) score one to three “no skills” in the four domains; and individuals with “no digital skills” score “no skills” in all four domains. In other words, they report to have no activities performed in all four domains, despite indicating that they used the internet at least once during last three months."

Page 5: inserted "Households and individuals were sampled through quota sampling methods (n = 1; Germany), one-stage sampling techniques (n = 2; Malta and Lithuania), or probability sampling (n = 25; the remaining 24 EU Member States and the UK) [[25]](https://www.zotero.org/google-docs/?k9UU6M). The exact means through which households or individuals were recruited differed per EU Member State (see 'Methodological Manuals' [[25]](https://www.zotero.org/google-docs/?k9UU6M)."

Page 5: inserted "Therefore, this study operates under the assumption that the findings of this study are an accurate reflection of the real situation among adults aged 16-74 in the European Union."

**p.4 First paragraph in "Variable Selection" is unclear**

Thank you for this comment. We have adapted the text to provide better clarification.

In-text edits:

Page 4: inserted "Digital skill levels are divided into “above basic”, “basic”, and “low.” The category “no digital skills” was removed from the analysis due to the unavailability of data."

**p.4 Sentence "Sub-stratification of education levels was possible by means of sex and three secondary age groups (16-24, 25-54, and 55-74 years)." is unclear.**

Thank you for this comment. We have revised and clarified the sentence to be better interpretable.

In-text edits:

Page 5: inserted "Education level was divided into three groups (low, medium, and high). Education level was further stratified by sex and three age groups (16-24, 25-54, and 55-74 years; data for the five age groups mentioned earlier were unavailable, hence the adjusted groups)."

**p.5 Info such as "Data was cleaned in R version 4.0.2, specifically using the ‘dplyr’ and ‘reshape2’ packages" is irrelevant. Provide the R code through a github repository instead.**

Thank you for this comment. While we disagree that naming the exact packages in the manuscript is irrelevant, we agree that providing the R code further enhanced the integrity of this article. We have amended the manuscript accordingly.

In-text edits:

Page 6: inserted "The exact code that was used with these packages is made openly available at https://github.com/robin-van-kessel/digitalskillsEurope/blob/8dae168c5b12fdec07431e1c11c125344bfd5e5e/Rcode."

**p.5 Fig.1 does not make much sense without any explanation**

Thank you for this comment. First, we realised Figure 1 was misplaced in the manuscript. It was meant to provide a rationale on why these exact variables were chosen to be included in the exploratory analysis. We have also added an explanation on the relevance of Figure 1 for this manuscript. We finally redrew Figure 1 to be more intuitive to read.

In-text edits:

Page 5: inserted "In order to properly select, understand, and interpret our findings and to infer possible consequences, we first established a directed acyclic graph showing theoretical pathways of how demographic variables affect the first and second levels of digital divides (Figure 1) [3,6]. Figure 1 allows us to take a theory-driven approach to the data analysis by outlining how variables are related in causing digital divides. It therefore allows us to extract relevant variables from the community survey to be included in the exploratory analysis: Internet access, digital skills, age, education level, employment, income, and urban status. Sex was only accessible through data on education level. Data on health status was not available"

**p.5 Descriptive Statistics: Given the sample size, I am puzzled by the high values of the standard deviations reported. As a quick approximation, for a simple random survey, the classical formula V(p) = (1-n/N)x(N/(N-1))xp(1-p)/n leads to estimated SD's several orders of magnitude smaller than those reported. Is there the possibility of a numerical mistake?**

Thank you for this comment. We have verified the standard deviation calculation in R using the sd() function, which resulted in the exact values reported in Table 1. The high values of standard deviations are indeed of particular interest, something that was insufficiently covered in the manuscript. As such, we added an interpretation of the high values of standard deviations.

In-text edits:

Page 7: inserted “What is particularly striking is the large spread of data (indicated by the large standard deviations), which can be attributed partially to the heterogeneity in how data was collected (a mix of households and individuals and sampling methods), but also highlights that it may be ill-advised to interpret the European Union as a homogenous region in terms of digital skills.”

**p.6 "Most individual respondents had above basic digital skills (34.65%)"**

**People tend to use "most" to mean anything over 50%**

Thank you for this comment. Even though we initially used ‘most’ as a relative term to indicate that this group had the highest prevalence compared to the other two measured skill groups, we understand why this formulation may cause confusion. As such, we have adapted the text according to your suggestion.

Page 6: “Overall, the highest proportion of people reported having…”

**Table 1 would be more efficiently placen in SM and replaced in the main text by a couple of figures.**

Thank you for this comment. We recognise that Table 1 includes a lot of data, some of which is not explicitly covered in the manuscript (i.e. data on devices used to access the Internet). At the same time, it would require numerous figures to accurately capture the data in Table 1 if it were moved to the SM. As such, after careful consideration, we decided to split Table 1 in two tables to make it easier to read. The half on what devices are used to access the internet is fully moved to the SM, while half that covers digital skills remains in the main manuscript.

**The whole data analysis reporting is labored and not easy to read. It should be better streamlined with an effort to focus on a few key results.**

Thank you for your comment. We have adjusted the results section to show a better narrative and interpretation of the data on top of the edits that were part of the earlier comments.

In-text edits:

Page 9: Inserted “The North and North-West are shown to have noticeably higher internet access rates compared to other European regions (94-98% compared to 89-91% in Central and South-West and 75-87% in South and East).”

Page 9: Inserted “Low income may also be associated with lower levels of internet access (33-96% compared to 65-100%; 83-100%; and 96-100% for the second, third, and fourth income quartile respectively).”

Page 10: Inserted “That being said, the development of above basic digital skills among younger people is inconsistent in Europe as indicated by the reported range (22-85%). Looking back at Figure 1, it is safe to assume that age needs to interact with other factors to reliably produce high digital skills.”

Page 10: Inserted “..., while rural areas report a similar spread of low digital skills (13-46%) as urban areas (11-43%).”

Page 10: Inserted “Finally, high income is associated with the development of higher digital skills (17-74%), although we have to note the wide spread here as well, implying that higher-income levels alone are not sufficient.”

**I found the discussion rather speculative, a bit verbose and somehow disconnected from the data analysis.**

Thank you for this comment. We have closely revisited our discussion to make it more informative, fluent, and more rooted in our own findings and the literature. We came to the conclusion that most paragraphs needed some thorough rounds of editing. One paragraph was removed entirely, which discussed the ‘third level of digital divides’ according to Kondratiev’s wave theory. After editing, this fell outside the scope of this article.

In-text edits:

Page 11: Inserted "...to examine the magnitude of the first..."

Page 12: Inserted "According to the directed acyclic graph in Figure 1, a plethora of factors has to work together in order for high levels of digital skills to develop. However, the reported spread of above basic digital skills may indicate that these factors work together inconsistently or insufficiently."

Page 12: Inserted "Next to the individual factors, there are also system-level factors that need to be considered, even though they have not been mapped by this particular dataset. As per Greenhalgh’s theory on the diffusion of innovation [[21]](https://www.zotero.org/google-docs/?Gm8e9J), it is important to consider whether – at a system level – there is a need and desire to innovate, whether countries possess the available resources and the necessary leadership to instigate and sustain change, and whether countries have a politico-cultural climate and population structure that facilitates the uptake of digital technologies."

Page 12: Inserted "...are more likely to be raised in a digitally-enabled environment [20,33,34]."

Page 12: Inserted "At the same time, the groups that could potentially benefit most from the uptake of digital technologies tend to be the groups that are faced with the biggest barriers to access [[6,12,27]](https://www.zotero.org/google-docs/?zxp6iK), whether that be due to socioeconomic status, health status, or other factors."

Page 13: Inserted "There are some limitations that have to be considered. Even though this article is underpinned by a large, representative data sample, we have to be mindful of differences among..."

**The final sentence "Europe is still unfit to sustainably host a digital society and doing so would exacerbate in- and cross-country inequalities" does not seem well motivated by the main findings and unduly worrisome.**

Thank you for this comment. We have amended the final sentence to be more in line with the findings and implications of this manuscript.

In-text edits:

Page 15: Inserted "Europe cannot be considered a homogenous zone in terms of internet access or digital skills and pursuing a complete digital transition would currently exacerbate in- and cross-country inequalities."
